# Safety during interhospital helicopter transfer of ventilated COVID-19 patients. No clinical relevant changes in vital signs including non-invasive cardiac output

**DOI:** 10.1186/s12931-022-02177-5

**Published:** 2022-09-19

**Authors:** Cornelis Slagt, Eduard Johannes Spoelder, Marijn Cornelia Theresia Tacken, Maartje Frijlink, Sjoerd Servaas, Guus Leijte, Lucas Theodorus van Eijk, Geert Jan van Geffen

**Affiliations:** 1 Helicopter Emergency Medical Service Lifeliner 3 and 5, Nijmegen, The Netherlands; 2 Department of Anesthesiology, Pain and Palliative Medicine, Radboud University Medical Centre, Geert Grooteplein Zuid 10, Huispost 717, Route 714, Postbus 9101, 6500 HB Nijmegen, The Netherlands

**Keywords:** Critical care, COVID-19, Noninvasive hemodynamic monitoring, Electrical cardiometry, Vital signs, Cardiac output. Helicopter Emergency Medical Service (HEMS), Interhospital helicopter transfer

## Abstract

**Background:**

During the COVID-19 pandemic in The Netherlands, critically ill ventilated COVID-19 patients were transferred not only between hospitals by ambulance but also by the Helicopter Emergency Medical Service (HEMS). To date, little is known about the physiological impact of helicopter transport on critically ill patients and COVID-19 patients in particular. This study was conducted to explore the impact of inter-hospital helicopter transfer on vital signs of mechanically ventilated patients with severe COVID-19, with special focus on take-off, midflight, and landing.

**Methods:**

All ventilated critically ill COVID-19 patients who were transported between April 2020 and June 2021 by the Dutch ‘Lifeliner 5’ HEMS team and who were fully monitored, including noninvasive cardiac output, were included in this study. Three 10-min timeframes (take-off, midflight and landing) were defined for analysis. Continuous data on the vital parameters heart rate, peripheral oxygen saturation, arterial blood pressure, end-tidal CO_2_ and noninvasive cardiac output using electrical cardiometry were collected and stored at 1-min intervals. Data were analyzed for differences over time within the timeframes using one-way analysis of variance. Significant differences were checked for clinical relevance.

**Results:**

Ninety-eight patients were included in the analysis. During take-off, an increase was noticed in cardiac output (from 6.7 to 8.2 L min^−1^; P < 0.0001), which was determined by a decrease in systemic vascular resistance (from 1071 to 739 dyne·s·cm^−5^, P < 0.0001) accompanied by an increase in stroke volume (from 88.8 to 113.7 mL, P < 0.0001). Other parameters were unchanged during take-off and mid-flight. During landing, cardiac output and stroke volume slightly decreased (from 8.0 to 6.8 L min^−1^, P < 0.0001 and from 110.1 to 84.4 mL, P < 0.0001, respectively), and total systemic vascular resistance increased (P < 0.0001). Though statistically significant, the found changes were small and not clinically relevant to the medical status of the patients as judged by the attending physicians.

**Conclusions:**

Interhospital helicopter transfer of ventilated intensive care patients with COVID-19 can be performed safely and does not result in clinically relevant changes in vital signs.

**Supplementary Information:**

The online version contains supplementary material available at 10.1186/s12931-022-02177-5.

## Background

During peak coronavirus-2 (SARS-CoV-2) spread, healthcare systems around the world were overwhelmed, with patients suffering from respiratory failure, many of whom needed hospitalization for ventilatory support. Distinctive of the COVID-19 outbreak was the emergence of regional hotspots, with large numbers of patients requiring intensive care (IC) admittance due to acute respiratory failure [1]. Severe COVID-19 presents with progressive dyspnea and often with hypoxemia [2, 3]. COVID-19 lung disease resembles ARDS and may rapidly progress to multiorgan failure [4]. Approximately 5% of severe COVID-19 patients require intubation and ventilation [5].

Before the COVID-19 pandemic, inter-hospital ICU transfer was performed with mobile intensive care units (MICUs) and specialized ambulances equipped as an ICU. When a local IC bed capacity problem arose, it was resolved by transferring patients regionally. Unfortunately, during the pandemic, the regional ICU capacity was insufficient at several hotspots in the Netherlands. Anticipating an increase in long-distance transfers, the team of Helicopter Emergency Medical Service (HEMS) Lifeliner 3 of the Radboud University Medical Center received permission from the Ministry of Health, Wellbeing and Sports to organize and operate the first ICU-equipped helicopter (Lifeliner 5) for transferring and redistributing critically ill COVID-19 patients in the Netherlands. This operation was initiated in cooperation with the Medical Air Assistance subdivision of the Royal Dutch Touring Club. Our group has described that helicopter transport of COVID-19 ventilated ICU patients is feasible and safe for patients and personnel [6].

However, it has been acknowledged that (long distance) transfers of ICU patients (inter-, intra- and prehospital) bear the risk of complications. Most of the (severe) complications are thought to be reflected by changes in vital signs [7–10]. With regard to helicopter transportation, specific concerns have been raised with regard to the effects of barometric pressure variations and acceleration/deceleration forces during take-off and landing [11]. Standard reports of vital signs during helicopter transport of ICU patients is lacking. Therefore, the goal of this study was to analyze the impact of interhospital helicopter transfer on vital signs of mechanically ventilated patients with COVID-19. Special focus on differences between take-off, mid-flight, and landing.

## Methods

In this prospective observational study, mechanically ventilated COVID-19 ICU patients who were monitored, including noninvasive cardiac output (CO), were included from the end of the first COVID-19 outburst in April 2020 throughout the end of the second outburst in June 2021. This study was assessed by the medical ethical committee Arnhem-Nijmegen, the Netherlands (identifier 2021–7313). This study was carried out in accordance with the applicable legislation and policy rules. The committee waived the need for informed consent. The study was registered at www.trialregister.nl (identifier NL9307).

### Data collection

Before monitoring started, monitoring devices were synchronized with the helicopter set-time and date. Three 10 min timeframes were defined for analysis. The first timeframe started with the actual take-off. The second timeframe represented midflight, and the third timeframe ended with the landing (touchdown). Data were extracted from the documented flight reports in the Operational Registration and Crew Administration data system. Continuous vital parameter data, including heart rate (HR), peripheral oxygen saturation (SpO_2_), arterial blood pressure (IAP), systolic, diastolic and mean arterial blood pressure (SBP, DBP and MAP), and end tidal CO_2_ (et-CO_2_), were collected for every patient during helicopter transfer using a Corpuls 3 monitor (Corpuls^®^ Benelux, Hellevoetsluis, The Netherlands).

### Noninvasive cardiac output measurement by electrical cardiometry

As cardiac involvement of SARS-CoV-2 was suggested during the first COVID-19 outburst [12], we expanded our standard monitoring in the second outburst with CO measurements. Cardiac Output data during IC helicopter transports is not available in the literature, let alone CO data during helicopter transfers of ventilated COVID-19 patients. Each CO measuring device has its own limitations. Invasive devices are linked to more complications. Choosing the right measurement method is complex and is determined by many factors including: patient, patient's illness, the environment of use, user experience and risk of complications [13]. Previously, our HEMS team had used the non-invasive CO monitor (ICON^®^ monitor, Osypka Medical GmbH, Germany) measuring device in the pre hospital setting. It is a practical easy to use, handheld device independent on IV access, in contrast to most invasive devices [14]. Because of the above we used the same noninvasive CO measuring device using electrical cardiometry (EC). Patient were connected to the device. Doctors and nurses were trained in the use of the noninvasive CO monitoring device (ICON^®^ monitor, Osypka Medical GmbH, Germany). According to the manufacturer’s instructions 4 skin sensors of the EC device were connected to the patient in the outplacing ICU and disconnected from the device at the arrival of the receiving ICU. This allowed continuous measurement of changes in thoracic electrical conductivity in response to a low amplitude, high frequency electrical current. Filtering techniques isolate changes in conductivity created by the circulatory system, which is mainly determined by blood in the aorta and its change in conductivity when subjected to pulsatile blood flow before and after aortic valve opening. This is used to derive the peak aortic acceleration (ACC) and left ventricle ejection time (LVET). Stroke volume (SV) is calculated using patient characteristics (gender, age, length, body weight), ACC and LVET [15, 16]. Further details of the device are described by Bernstein and colleagues [15, 17]. As central venous pressure was not recorded, total systemic vascular resistance (TSVR) was calculated using the formula MAP/CO*80 (dyne·s·cm^−5^) as described in the literature [18]. Vital signs, including noninvasive CO data, were recorded at 1-min intervals.

### Adverse events

Major adverse events during inter-hospital transfer were defined as cardiac or respiratory arrest, pneumothorax or seizure. Minor adverse events were defined as *new* undesirable changes in vital signs, outside the limits of the threshold value, during take-off, midflight or landing. Standard target values of hemodynamics for patients in the ICU are well defined; however, cut-off values for vital signs that are considered to be causing harm are less well defined. In this study, cut-off values were defined according to the available literature, taking into account that this study included ventilated critically ill patients with COVID-19. We defined the following thresholds: MAP < 60 and < 65 or > 120 mmHg; SpO_2_ < 88 and 90%; et-CO_2_ < 25 or > 55 mmHg; HR < 50 and > 100 bpm requiring treatment [19–23].

### Statistical analysis

For the analyses and comparison of vital parameters, the aforementioned 10-min window of patient data at take-off, mid-flight and landing was used. The resolution for vital sign data was set at 1 min. In the case of missing data, only timeframes with ≥ 6 data points were eligible for analysis. Missing data were interpolated with the mean of previous and next data points or extrapolated with the last observation carried forward. Data were assessed for normal distribution using the Kolmogorov–Smirnov test and presented accordingly (normally distributed data with mean ± standard deviation and not normally distributed data with median [interquartile range]). For every vital parameter, differences over time within the group were analysed using mixed effect analysis and Dunnett’s multiple comparison test for post hoc analysis. Not normally distributed data were log10(x) transformed before mixed effect analysis. A P-value < 0.05 was considered statistically significant. All statistically significant differences were checked for clinical relevance [19–23]. Data were analysed using the appropriate tests in GraphPad Prism version 9.4.0 (GraphPad software, San Diego, USA).

## Results

### Patient characteristics

Ninety-eight patients were included in this analysis. Patient characteristics are shown in Table 1. Most patients were deeply sedated, receiving one or two (49%) sedatives (midazolam and propofol) opioids 93% and rocuronium 46%. Bolus medication (midazolam, opioid, propofol, ketamine, nor-epinephrine or phenylephrine) was given in 70 patients to facilitate safe transportation and reduce oxygen consumption and carbon dioxide production. Eighty-four patients received continuous norepinephrine to support hemodynamics.

**Table 1:** Patient characteristics

Gender M/F	69/29
Age (year)	62.3 ± 11.5
Weight (kg)	91.6 ± 16.7
Height (cm)	175 ± 9.4
Body Mass Index (kg m^−2^)	30.3 ± 6.0
Ventilation mode	
PCV	80 (82%)
PEEP (cmH_2_O)	12.4 ± 2.2
PC (cmH_2_O)	14.3 ± 2.7
VCV	10 (10%)
PEEP (cmH_2_O)	12.3 ± 2.6
TV (ml)	407 ± 42
PSV	8 (8%)
PEEP (cmH_2_O)	10.7 ± 2.3
PS (cmH_2_O)	8.7 ± 3.6
Trachea cannula	4 (4)
Inspired oxygen (%)	52 ± 12
Flight time (min)	56 ± 16
Range (min)	35–149
Total flight time (min)	5497
Flight distance (km)	156 ± 46
Range (km)	71–272
Total distance (km)	15,314
Sedation	
Midazolam	26 (27%)
Propofol	24 (25%)
Midazolam + Propofol	48 (49%)
Opoids	91 (93%)
Rocuronium	45 (46%)
Bolus number	
Sedation	70
Opiods	20
Rocuronium	45
Vasoactive	14
Nor epinephrine	82 (84%)
Mean dose ± SD (µg/kg/min)	0.07 ± 0.05

Data are expressed as numbers, percentages, and mean ± standard deviation were appropriate

*PCV* pressure control ventilation, *PEEP* positive end expiratory pressure, *PC* pressure control, *VCV* volume control ventilation, *TV* tidal volume, *PSV* pressure support ventilation, *PS* pressure support

### Data

All continuous parameters were recorded at a 1-min interval during all timeframes. Technical monitoring problems led to the loss of 281 data points (1.6% of total). For CO, SV and TSVR, a total of 522 timeframes were available for analysis (59.1% of total). Available timeframes were equally divided between take-off, midflight and landing.

### No profound changes in vital parameters during take-off, midflight and landing

**Fig. 1:**
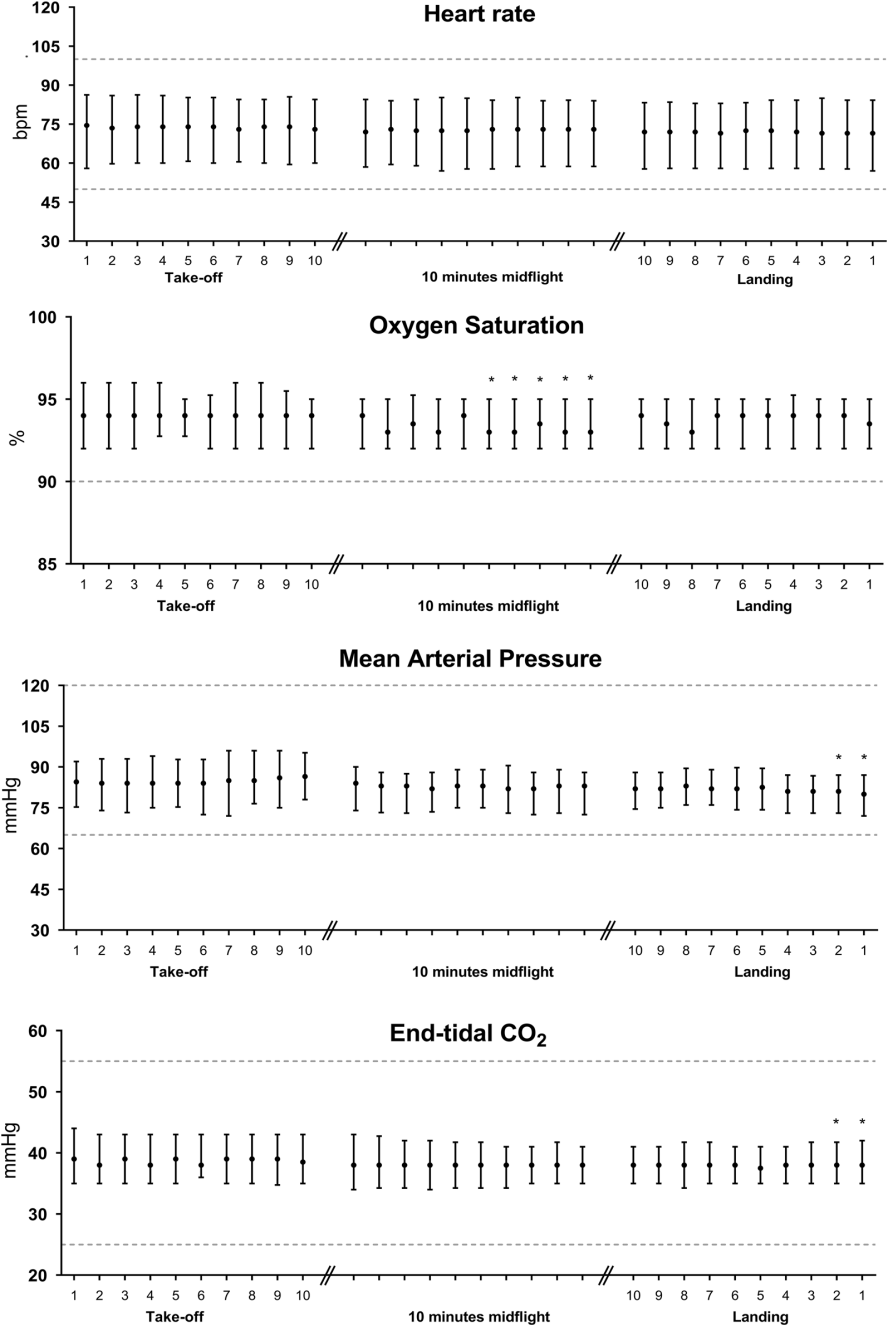
Vital signs during take-off, midflight and landing. Data are expressed as median and interquartile range. *Bpm *beats per minute. *P < 0.05 measured using Dunnett’s multiple comparison test. Consecutive measurements in each timeframe are compared to the first measurement in that timeframe

**Fig. 2:**
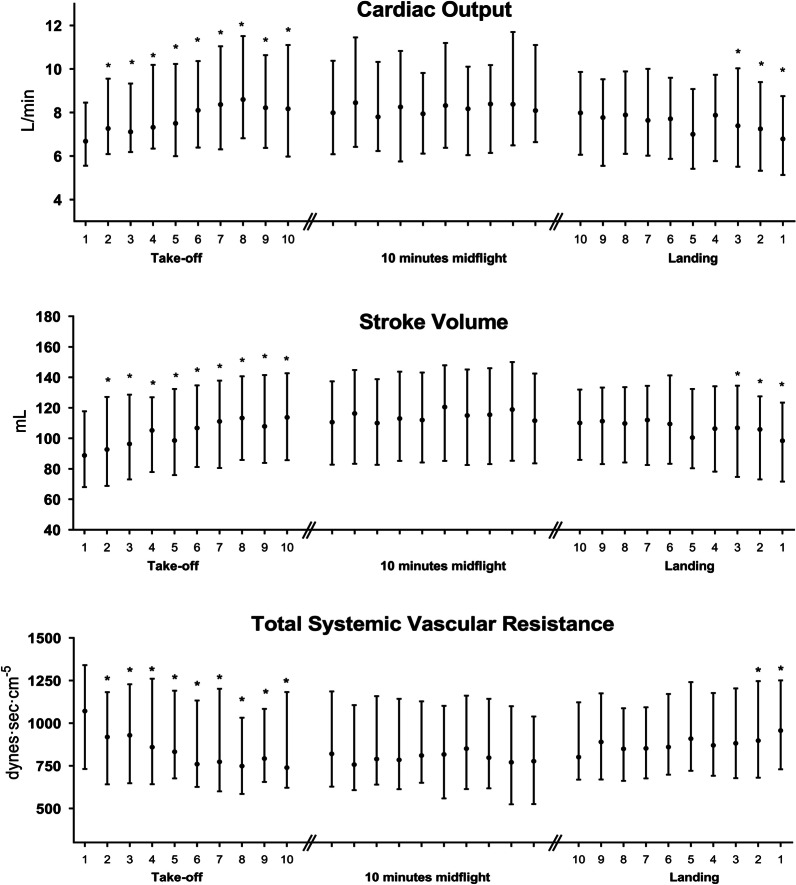
Noninvasive cardiac output data during take-off, midflight and landing. Data are expressed as median and interquartile range. *L/min* liter per minute, *mL *milliliter. *P < 0.05 measured using Dunnett’s multiple comparison test. Consecutive measurements in each timeframe are compared to the first measurement in that timeframe

Overall, no profoundly clinically relevant changes were observed in vital signs during inter-hospital helicopter transfer of ventilated COVID-19 patients (Figs. 1, 2; Additional file 1: Fig. S3; Table 2). Although there were several statistically significant changes within the timeframes (Table 2), these were not deemed clinically relevant, as most values were within normal ranges. [19–23].

**Table 2:** Difference in vital signs during take-off, midflight and landing timeframes

	Mixed effect analysis on log transformed data		
	Take-off	Midflight	Landing
SpO_2_	0.58	< 0.0001	0.84
HR	0.62	0.52	0.57
SVR	< 0.0001	0.38	< 0.0001
CO	< 0.0001	0.25	< 0.0001
SV	< 0.0001	0.61	< 0.0001
MAP	0.55	0.81	< 0.0001
SBP	0.90	0.90	< 0.0001
DBP	0.58	0.27	0.30
CO_2_	0.52	0.40	< 0.0001

*SpO*_*2*_ peripheral venous saturation, *HR* heart rate, *SVR* systemic vascular resistance, *CO* cardiac output, *SV* stroke volume, *MAP* mean arterial pressure, *SBP* systolic blood pressure, *DBP* diastolic blood pressure, *CO*_*2*_ end tidal carbon dioxide

*P < 0.05 measured using mixed effects analysis

During take-off, there were no significant changes in basic vital signs, such as HR, CO_2_, SpO_2_ and IAP. Mid-flight was the most stable timeframe with respect to vital signs; only for SpO_2_ was a difference over time seen. In the last minute before landing MAP and et-CO_2_ show significant changes.

During take-off, an increase in stroke volume from 88.8 [68.0–117.7] to 113.7 [85.6–142.7] mL; P < 0.0001 was found. This led to an increase in cardiac output from 6.7 [5.6–8.5] to 8.0 [6.1–10.4] L min^−1^; P < 0.0001. With minimal changes in MAP, this led to a decrease in total systemic vascular resistance from 1071 [732–1341] to 739 [622–1182] dyne·s·cm^−5^; P < 0.0001. During landing we see the opposite effects. SV decreases from 110.1 [85.9–131.9] to 98.4 [71.6–123.4] P < 0.0001. TSVR changes from 801 [669–1122] to 957 [730–1251] dyne·s cm^−5^; P < 0.0001. CO changes from 8.0 [6.1–9.9] to 6.8 [5.1–8.8] L min^−1^; P < 0.0001. Reaching its take-off values (take-off vs landing timeframe; P = 0.80).

### Adverse events

No major adverse events took place during the transfers. Minor adverse events are depicted in Table 3. The most frequently observed adverse event was a new MAP < 65 mmHg, and only 2 events lasted longer than 5 min. None of the MAP < 60 mmHg events lasted longer than 5 min.

**Table 3:** Minor adverse events

	Take-off	Midflight	Landing	Total
MAP < 65 mmHg	11	9	9	29
< 60 mmHg	3	1	3	7
> 120 mmHg	4	2	–	6
SpO_2_ < 90%	3	–	2	5
< 88%	1	1	2	4
Et-CO_2_ < 25 mmHg	–	1	–	1
> 55 mmHg	1	2	1	4
HR* < 50 bpm	–	–	–	–
> 100 bpm	–	–	–	–
Total	23	16	17	56

*SpO*_*2*_ peripheral venous saturation, HR* heart rate requiring treatment, *MAP* mean arterial pressure, *et-CO*_*2*_ end tidal carbon dioxide

## Discussion

The current observational study showed that inter-hospital helicopter transport of mechanically ventilated COVID-19 patients can be performed safely. No clinically relevant changes in basic vital signs as heart rate, oxygen saturation, blood pressure, et-CO_2_ or non-invasive hemodynamic monitoring as SV and CO were observed during take-off, mid-flight or landing.

Several studies have described the organization and quality of intensive care transport [24–26]; however, most data are obtained from road transfers using MICUs, and data about COVID-19 transports are limited [22, 27, 28]. In the early days, Waddell and colleagues described the impact of ambulance transport on the vital signs of critically ill patients [29]. They documented considerable variation in hemodynamic responses to transport between patients. Increased mortality was found in individuals who developed hypertension or hypotension compared to patients with no change or delayed hypotension. More recent publications did not find an increased mortality after interhospital transfer by ambulance or MICU [10, 30, 31] even in COVID-19 patients [32]. But interhospital transfer of critically ill patients is not without risks [33, 34]. Unfortunately little is known in relation with changes in vital signs in combination with helicopter transfer.

To minimize complications it is important that the HEMS IC transport is imbedded within regular care [35]. A thorough preparation of the transfer begins with a daily check of all medical equipment, oxygen supply, medication, helicopter safety checks and weather conditions. Consultation with out placing and receiving intensivists (know your patient). Prepare the patient; minimize pumps and infusing systems, empty all collection bags (urine, gastric content) or drains. Hereafter the actual planning can take place based on the requirements of the patient. Logistic challenges are discussed and resolved minimizing secondary transfers. The weather conditions and flight path for this transfer are checked for problems with special focus on take-off and landing platforms. Fuel needed or refueling options. At the outplacing IC handover by intensivist and don’t forget medical documents. Ensure a calm and structural handover and transfer of the patient to your own branch. First the patient, hereafter syringe pumps, urinary catheter, drains and finally the ventilation. Check all connections. Use a structural approach to load and unload the patient from the helicopter. Continue patient care and patient comfort during the flight. Expect the unexpected and have an open team communication [26–28].

Malagon et al. investigated noninvasive cardiac output during fixed wing air ambulance repatriation using suprasternal Doppler [36]. Six medical crew members and 7 patients were assessed on the ground, during take-off, in the air and during descend. They found a statistically significant increase in CO and SV during take-off. A possible explanation was the combination of G-forces and the occurrence of mild hypoxia releasing NO mediators. However, similar to our findings, their changes were without clinical relevance. Our patients were on mechanical ventilation and thus received supplemental oxygen. The cruising altitude of our IC-helicopter was between 800 and 1200 feet. Taken together, relative hypoxia was not a contributing factor during our transport. Gravitational forces are limited during medical helicopter transport. During take-off and landing, acceleration/deceleration occurs in a 1–2 min window after take-off and before landing. As HR was not influenced (Fig. 1) during the three time frames an increase in SV is be responsible for the increase in CO. During the entire flight, vibration of the helicopter could induce pooling of venous blood, thereby changing cardiac preload and influencing SV and CO. Most importantly, during the first minute of actual lift off, the patient is positioned in a head-up position; hereafter, the patient is positioned in a slight head-down position during the entire flight. This position increases venous return and could be responsible for the observed increase in SV and CO. The observed increase in SV and CO led to an increase in oxygen delivery, which is anything but an adverse event [19, 23].

Although noninvasive CO measurements have been performed before in the prehospital HEMS setting [14, 37], limited data are available on CO measurements during IC-helicopter transports, let alone COVID-19 transfers [14, 37]. Our study is the first to attempt CO measurements during interhospital helicopter transfer in ventilated IC patients with COVID-19. In all 98 patients, noninvasive CO measurements were performed. However, 59.6% of the timeframes were available for analysis due to missing data. As ICON measures changes in electrical conductivity within the thorax in response to a low amplitude, high-frequency electrical current, the signal could plausibly be influenced by vibrations or movement generated during flight [14, 16, 17]. In previous work, ICON hemodynamic monitoring in the prehospital emergency medical setting showed that 23.6% of all measurements were lost due to interference with the signal. In that study, five out of 50 patients were transferred by helicopter, and all had good measurements [14].

With only 1.65% missing data of the conventional vital sign parameters, a robust data set was created to evaluate the impact of interhospital helicopter transport. Although there is no consensus for strict target parameters, Alhazzani et al. provided guidelines for the general management of critically ill adults suffering from COVID-19 [19]. They suggested an SpO_2_ ≥ 90% in patients with acute hypoxemic respiratory failure. A target MAP of 60–65 mmHg is suggested, often requiring vasopressor support due to the high ventilation pressures needed to optimize ventilation and to counteract deeply sedated patients [3, 19]. In accordance, 84% of our patients needed vasopressor therapy to support the circulation. Extra sedation was given to optimize ventilation, reduce oxygen consumption, decrease carbon dioxide production and reduce the stresses of flight, which could have had an impact on the measured vital signs. No major adverse events occurred, and only limited minor adverse events were recorded in our study, in which we flew more than 15,000 km with COVID-19 patients.

This study has limitations. We conducted research in a relatively new patient population in a single-centre study. Therefore, extrapolation of these results to other intensive care patients may be limited. The use of noninvasive hemodynamic monitoring could be seen as a limitation. The EC measurements are sensitive for disturbance. During our IC helicopter transport 40.4% of the noninvasive hemodynamic data were missing but there were no complications due to the CO measurements but still 5220 data points were available for analysis. The threshold values taken in this study may not be acceptable to others. Finally, our study lacks outcome data. Interhospital transfers do bear risks for ICU patients. Recently outcome data has become available showing minimal or no impact on patient wellbeing [31–34]. The complexity of this operation in a time were all healthcare workers were under maximal physical and mental strain and the lack of permission from the medical ethics committee to collect these data made it not possible to register outcome data.

## Conclusion

Interhospital helicopter transport of ventilated intensive care patients with COVID-19 can be performed safely. Although some significant changes in basic vital signs and noninvasive hemodynamic measurements were found during take-off, mid-flight and landing, these were not clinically relevant. All vital signs are merely within the normal ranges of vital signs seen during intensive care treatment.

## Supplementary Information

**Additional file 1: Fig. S1**. Systolic and diastolic blood pressure during take-off, midflight and landing. Data are expressed as median and interquartile range. *P < 0.05 measured using Dunnett’s multiple comparison test. Consecutive measurements in each timeframe are compared to the first measurement in that timeframe.

## Data Availability

The datasets used and/or analysed during the current study are available from the corresponding author on reasonable request.
