# Supplementary figures and images for: Safety during interhospital helicopter transfer of ventilated COVID-19 patients. No clinical relevant changes in vital signs including non-invasive cardiac output
Source: Respir Res. 2022 Sep 19;23:256. doi: 10.1186/s12931-022-02177-5 (PMC9484339; doi:10.1186/s12931-022-02177-5)

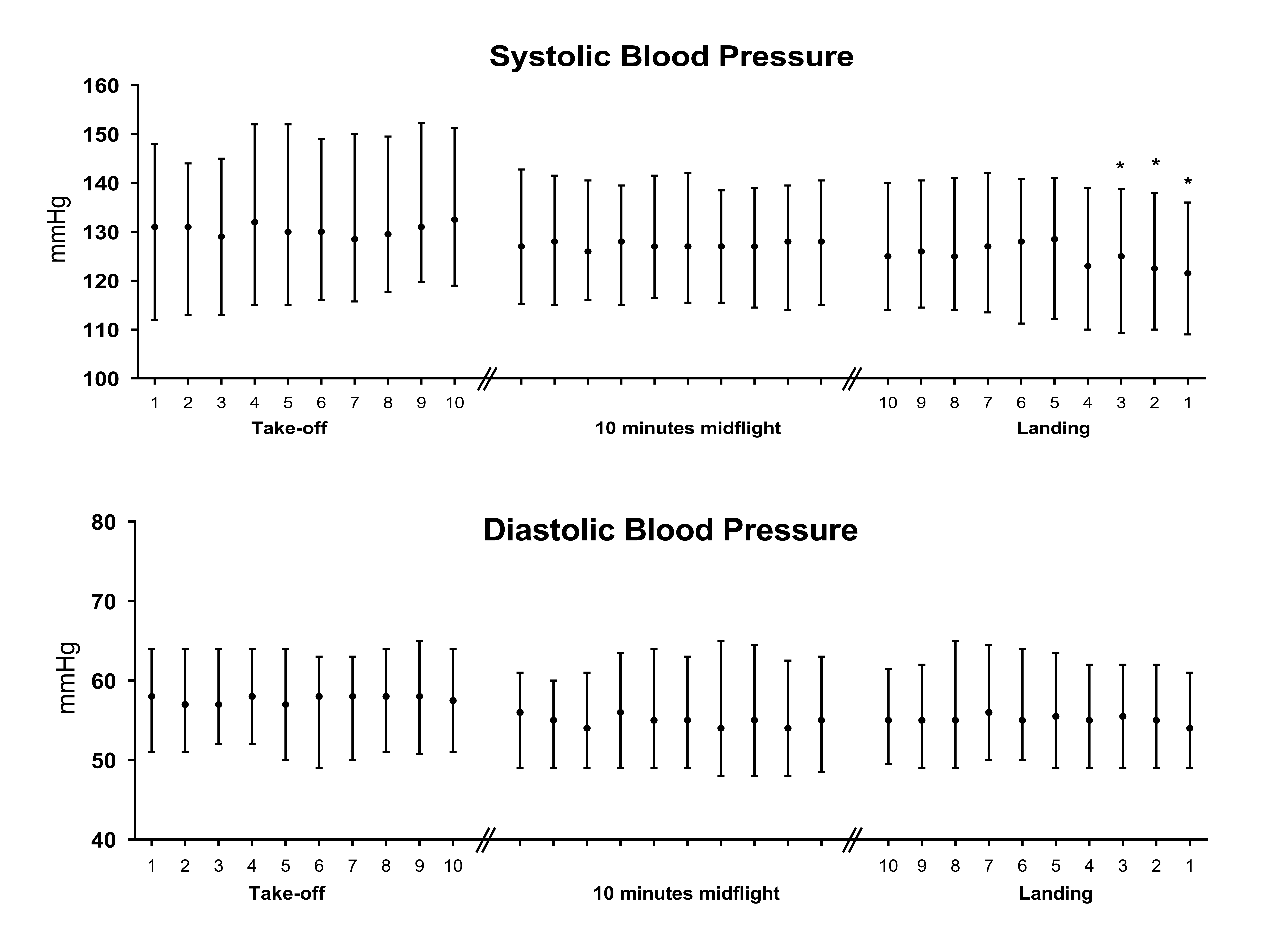

Supplement: Supplementary file 1 — Additional file 1: Fig. S1. Systolic and diastolic blood pressure during take-off, midflight and landing. Data are expressed as median and interquartile range. *P < 0.05 measured using Dunnett’s multiple comparison test. Consecutive measurements in each timeframe are compared to the first measurement in that timeframe. [file 12931_2022_2177_MOESM1_ESM.tif]
